# Bacteria-dependent modulation of immune responses in the bovine udder

**DOI:** 10.1186/s13567-026-01754-6

**Published:** 2026-04-10

**Authors:** Alicja Krysmann, Ian Woolsey, Vinicius da Silva Duarte, Christian Kranjec, Davide Porcellato, Preben Boysen

**Affiliations:** 1 Faculty of Chemistry, Biotechnology and Food Science, Norwegian University of Life Sciences, Ås, Norway; 2 Faculty of Veterinary Medicine, Norwegian University of Life Sciences, Ås, Norway; 3 Department of Molecular Oncology, Institute for Cancer Research, Oslo University Hospital, Oslo, Norway

**Keywords:** Mastitis, host, pathogen, microbiome, immune response, proteomic, bovine

## Abstract

Subclinical mastitis remains difficult to characterize due to the complex interplay between pathogens, local microbiota, and host immune responses. The aim of this study was to investigate how different bacterial species detected in the hindmilk influenced bovine udder immune response and its regulation. Quarter-level hind milk samples were collected from 24 healthy Norwegian Red cows before drying off and through a next full lactation period. Somatic cell populations, cytokines and host-proteome were assessed and combined with the presence of different pathogens. Quarters harboring *Staphylococcus aureus* and *Streptococcus* spp. showed significantly elevated somatic cell counts and inflammation associated patterns of somatic cell populations, while those with minor pathogens exhibited lower counts, particularly fewer granulocytes. Cytokines profiling revealed suppressed levels of IFN-γ, IL-10 and TNF-α in the presence of *Staphylococcus chromogenes* in contrast to the intensive inflammatory activity associated with *S. aureus*. Weighted gene co-expression network analysis of the host proteome identified two modules of proteins that were negatively correlated with increasing somatic cells count and several modules with strong positive correlation with the SCC. At protein level, we detected 67 proteins differentially expressed based on the pathogen present in the hindmilk. Of these, 19 were linked to immune system functions such as TLR2 and lactoferrin. Our study provides insights into host response in bovine mastitis and reveals a notable lack of interaction between *S. chromogenes* and the host body compared to an intense immune response in the presence of other pathogens such as *S. aureus*.

**Supplementary Information:**

The online version contains supplementary material available at 10.1186/s13567-026-01754-6.

## Introduction

Mastitis is a major challenge in the dairy industry, causing economic losses, affecting animal welfare representing a leading cause of antibiotic treatment in the dairy industry [1]. This inflammatory disease of the mammary gland is mainly caused by intramammary infection (IMI), though mechanical damage to the udder, such as being hit or striking a barrier, can also lead to inflammation. Bacterial proliferation in the gland triggers the host immune response, leading to inflammation. Bacterial growth can cause tissue damage to the mammary gland, which can significantly increase inflammation.

Several pathogens can cause IMI, with incidence influenced by geography, farm management, and host genetics. The most common are *Staphylococcus aureus* (*S. aureus*), non-*aureus* staphylococci and mammaliicocci (NASM), *Streptococcus uberis* (*S. uberis*), *Streptococcus dysgalactiae* (*S. dysgalactiae*), and *Corynebacterium bovis* (*C. bovis*) [2–5]. Mastitis presents in two main forms: clinical, with visible signs such as swelling or fever, and subclinical, which lacks external symptoms but is characterized by reduced milk yield and quality, elevated somatic cell count (SCC), pathogen presence in milk. Subclinical mastitis is challenging to manage because detection requires laboratory testing, and affected cows may continue producing milk while shedding bacteria [3, 6, 7].

The immune response to IMI pathogens is a complex interplay of innate and adaptive immune systems. The first line consists of mechanical barriers such as teat apex and teat canal, as well as the Mammary Epithelial Cells (MECs) [4, 8, 9]. Secretory MECs actively participate in innate immune system activators, by expressing on their surface Toll-like receptors (TLRs) that recognize Pathogen Associated Molecular Patterns (PAMPs), triggering signaling pathways that introduce pro- and anti-inflammatory cytokines. Proinflammatory cytokines attract immune system cells while epithelial cells secrete antimicrobial proteins like lactoferrin. Effector cells (called later somatic cells), primary leucocytes, the majority of them being neutrophils, migrate to the site of infection [8, 9]. The rapid recruitment of somatic cells leads to an increase in SCC, which are naturally present in low numbers (below 200 000) in the milk of healthy cows [2]. Increase of SCC together with pathogen detection leads to the determination of the correct treatment in dairy cattle. Studies have shown that SCC elevation is not equal in all cases and can depend on the bacterial species causing IMI [10, 11]. This highlights the importance of understanding pathogen associated patterns of immune response in mastitis.

Among mastitis pathogens, *S. aureus* is a globally prevalent cause of clinical and subclinical mastitis, often contagious within herds, persistent in the mammary gland, and associated with long-term SCC elevation and recurrent infection [9]. NASM, frequently isolated in subclinical mastitis cases, typically cause milder immune responses, though strain-level variation has been reported, especially for *S. chromogenes* [12, 13]. *Corynebacterium bovis*, traditionally considered a minor pathogen, has recently attracted interest for its potential protective effect against infections with major pathogens such as *S. aureus* [14, 15].

Pathogen-specific variation in immune response has been long recognized, yet most studies have focused on a limited set of primary pathogens. Bannerman et al. [16] examined cytokine profiles following experimentally induced IMI with *Escherichia coli* (*E. coli*) and *S. aureus*, demonstrating pathogen-specific differences in immune activation. Similarly, Hagiwara et al. [17] investigated lactoferrin levels in milk, reporting elevated levels in samples positive for *S. aureus* or *Streptococcus* spp. compared with NASM or *C. bovis*. These studies, however, remain limited in scope, with a focus on a few pathogens or single immune markers and primarily utilizing one or two different methods.

The most extensively used non-invasive methods for assessment of immune responses include ELISA-based cytokines detection, often combined with total SCC and differential somatic cells count (DSCC) levels [18–21]. Recently proteomic analysis of somatic cells has been applied particularly in high—SCC samples from subclinical mastitis [19, 22]. However, no studies so far have investigated the proteome of somatic cells in association with a broad range of pathogens.

To address these gaps, we applied a multimethod, noninvasive approach to characterize the dairy cows’ immune response to a broad range of bacterial taxa. Our study integrates cytokine measurements, proteome profiling, and flow cytometry of somatic cells to provide new insights into the regulatory mechanisms shaping bovine immune responses in subclinical mastitis.

## Materials and methods

### Sample collection

Milk samples were collected from Norwegian Red cows selected from animals with no signs of sickness at the herd at “The Livestock Production Research Centre” at Norwegian University of Life Sciences. The 24 cows were selected from a list of 43 cows that were available at the University farm and that had calving in the period of December 2022 and February 23. Of the initial 43 cows, 8 cows were removed because they were included in another study (where some of them were fistulated) or under antibiotic treatment. From the remaining 35 cows, individuals were selected based on the number of lactations, aiming for 8 cows in their first lactation and 16 cows in their second or later lactation. This approach was used to cover the diversity of cows present at the farm. After selection, the cows were grouped in groups of 5 so that, on each sampling day, 20 samples were obtained and analyzed. The farm is managed according to Norwegian Food Safety Authority regulations, the cows are kept in freestalls lined with rubber mats and raw woodchips. The feed includes pellet food in amounts dependent on individual milk production of each cow and silage. Samples were collected at four timepoints in period between autumn 2022 and autumn 2023. Sampling occurred before the drying off period for a subset of 16 multiparous cows and continued throughout the entire subsequent lactation period for whole group of 24 cows. Samples were collected at three time points: at the beginning (early stage) in the middle and towards the end of lactation over 5-month period at equal intervals. Each cow was sampled on at least three separate occasions corresponding to these stages, while multiparous cows were sampled a total of four times. Total of 342 quarter levels samples were collected, all cows contributed samples from all four quarters, except for one cow that had one non-functional quarter (at three sampling points) and one cow that was removed from the study prior to the final sampling. Subsequent analysis included a smaller subset of this data. Milk (350 mL) was collected manually from each quarter separately at the end of normal milking following the “Procedure for Collecting Milk Samples” by the National Mastitis Council [23]. After detaching milking robot each teat was cleaned with iodine and 70% ethanol. Samples were collected aseptically into sterilized bottles. Samples were stored on ice and delivered to the laboratory within 2 h of collection, where samples were aseptically aliquoted into polypropylene tubes. Proteomics and flow cytometry samples were processed until the pellet step immediately after, whereas samples for cytokines analysis were stored at −20 °C.

### Microbiota characterization

A subset of 337 quarter level samples taken from the 24 cows were included in this part of the study. The microbiota of each quarter was investigated by 16S rRNA amplicon sequencing and shotgun metagenomics in a previous study [24]. For the purpose of this study, the amplicon sequencing data were retrieved, and the samples were classified based on the presence of known bacterial species or genres. The presence of a certain taxa was considered true if the relative abundance of sequence variance was over 30% of the total microbiota. Each sample was assigned to only one group, in case of sample having multiple sequence variance at over 30% of abundance, the most abundant variant was considered. All samples in which amplicon sequencing was performed were assigned to pathogen groups, samples with no sequence variance above 30% were considered negative. For data analysis and interpretation samples were grouped into pathogen groups based on their taxonomic similarity and clinical relevance of the detected species. For example, samples containing *Staphylococcus epidermidis* (*S. epidermidis*) were grouped into *S. epidermidis* group, and those with *S. chromogenes* to *S. chromogenes* group. To limit the number of groups created less frequently detected species like *Staphylococcus xylosus*, *Staphylococcus haemolyticus*, *Staphylococcus hominis*, and other *Staphylococcus* sp. were combined into a single “other NASM” group. A similar approach was applied when creating groups for *Corynebacterium* and *Streptococcu*s species. The level of bacteria and SCC was obtained using the BacSomatic instrument (FOSS, Hillerød, Denmark).

### Flow cytometry

#### Sample preparation

Fifty mL of milk was centrifuged at 400 *g* for 5 min at 4 °C, the fat layer and 40 mL of supernatant were removed. The cell pellet was resuspended in the remaining 10 mL of supernatant. Cold 1 × Dulbecco's Phosphate Buffered Saline (DPBS) was added (40 mL), and the sample was recentrifuged as before. Forty-five mL of supernatant were removed and in the remaining DPBS, the cell pellet was resuspended via agitation. Samples were strained through a 70 µm cell strainer (Avantor, PA, USA) into 50 mL polypropylene tubes and 5 mL of cold 1 × DPBS was added before another round of centrifugation (400 *g* for 5 min). The supernatant was removed, and three replicates of 300 µL were added to a 96-well U-bottom plate (Falcon, Corning, NY, USA). For large pellets 100 µL were used. Plates were spun at 800 × *g* for 1 min at 4 °C and the supernatant was removed.

#### Staining and flow cytometry analysis

Samples from all animals from a single sampling point were included on each plate, with one well corresponding to a quarter of each animal (four wells per animal per plate). All plates included 3 types of controls for each animal (one well per control per animal): unstained cells; cells only incubated with primary mononuclear antibodies and cells stained with only live/dead reagent per animal. The controls for each animal were taken from a randomly selected quarter. An additional control plate was included on two occasions with Fluorescence Minus One (FMO) (Additional file 1) and single controls from one animal selected at random at that sampling point. One well was used for each control, with milk from each quarter of that animal pooled in equal proportions. Reagents used in flow cytometry are listed in Table 1, all steps were performed on ice unless otherwise stated.

**Table 1:** **Reagents used in flow cytometry: primary antibodies and auxiliary reagents**

Antigen	Target species	Conjugate	Clone	Isotype (mouse)	Vendor	Catalogue no	Final conc. or dilution
*Primary antibodies*							
CD45	Ovine	biotin	1.11.1932	IgG1	BioRad	MCA2220B	2 µg/mL
CD14	Human	PE-Vio770	TÜK4	IgG2a	Miltenyi	130-113-149	1:50
MHC class II	Bovine	Alexa 647	IL-A21	IgG2a	Bio-Rad	MCA2445A647	1:50

Reagent	Conjugate	Vendor	Catalogue no	Final conc. or dilution
*Auxiliary reagents*				
Streptavidin	PerCP-Vio700	Miltenyi	130-106-795	1:200
Vybrant™ DyeCycle Violet		Invitrogen	V35003	5 µM
Zombie NIR™ Fixable Viability Kit		BioLegend	423106	1:500

**Figure 1:**
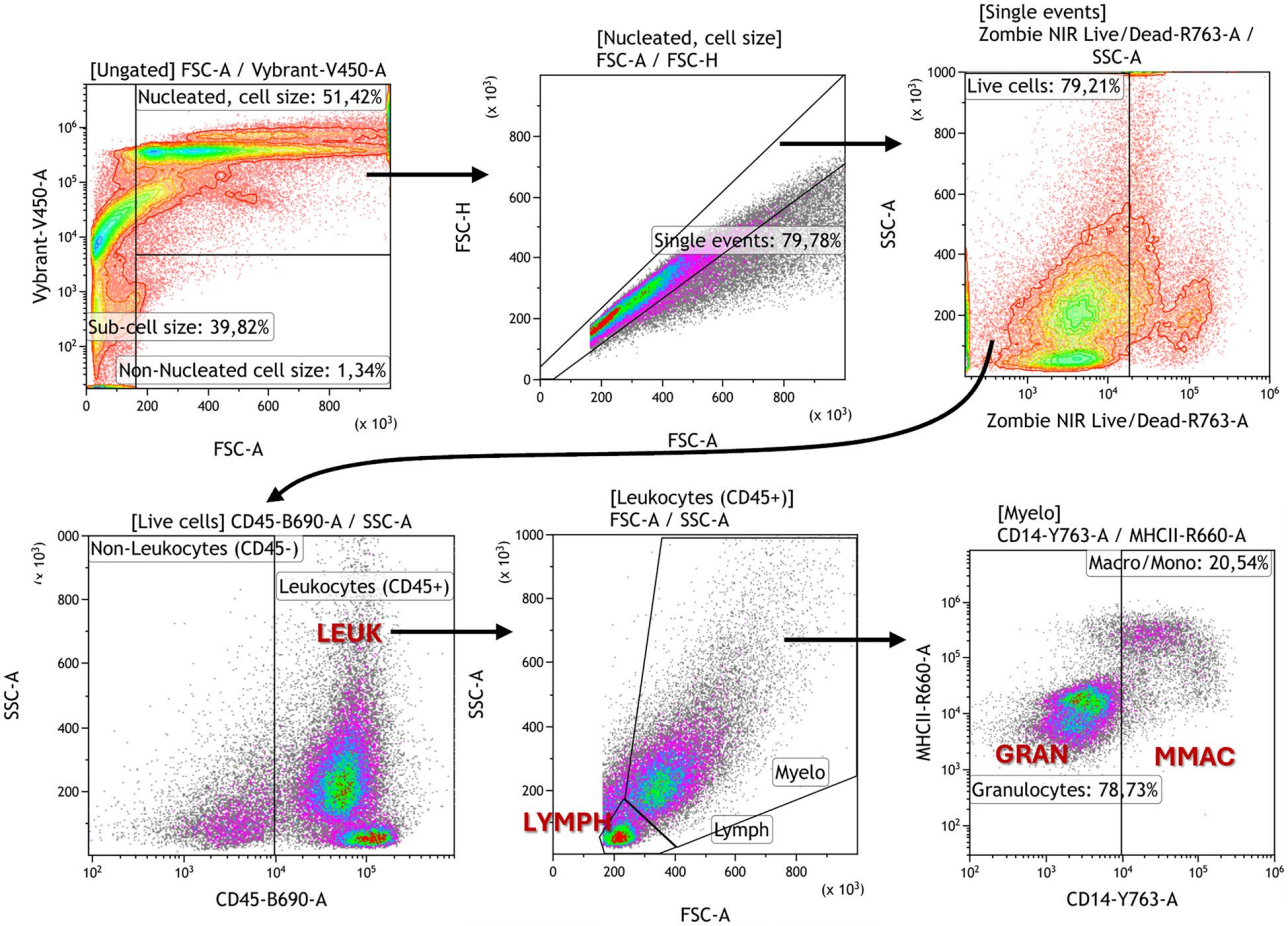
**Gating strategy for definition of cellular subpopulations by flow cytometry.** Axes: [Marker]—[Laser (V = violet; B = Blue; Y = yellow; R = Red)] [Emission filter]—[Signal type (A = Area)]. Red capitals refer to cell subset variables (LEUK—leukocytes, LYMPH—lymphocytes, GRAN—granulocytes, MMAC—monocytes and macrophages).

100 µL of Zombie NIR live/dead reagent (1:500) was added to wells, excluding non-stained controls, where 100 µL DPBS was added, and the plate was incubated at RT in the dark for 20 min. Cells were subsequently washed with 150–200 µL of DPBS buffer and centrifuged at 800*g* for 1 min at 4 °C; the supernatant was removed afterwards. Primary antibodies diluted in flow buffer (DPBS with 0,5% Bovine Serum Albumin (BioRad cat.no. 805090) and 10 mM NaN_3_ (Sigma-Aldrich)) were then added to the sample and control wells and incubated in the dark for 30 min. The samples were then washed twice with flow buffer. Secondary reagent was then added and incubated in the dark for 30 min followed by washing in 200 µL flow buffer. Cells were fixed by adding 100 µL of Intracellular Fixation Buffer (Invitrogen cat.no. FB001), pulse vortexed, and incubated for 10 min, followed by spinning down the plate and a single wash with HBSS (5% BSA). Finally, the cells were resuspended in 150 µL HBSS (5% BSA) for short-term storage at 4 °C. Immediately before flow cytometry reading, Vybrant DyeCycle Violet DNA stain (5 µM) was added, and then the samples were incubated at RT for 30 min. Lastly, the samples were filtered through a 70 µM nylon mesh and analyzed by flow cytometry without further washing steps. All samples were analyzed with a Cytoflex LX 4-laser (V-B-Y-R) flow cytometer (Beckman Coulter), and data were analyzed using Kaluza software v.2.3 (Beckman Coulter). Gating strategies for cell identification are shown in Figure 1.

### Cytokines analysis—multiplexing ELISA

Aliquoted 10 mL of milk samples were thawed and centrifuged at 8000 *g* for 10 min at 4 °C, the fat layer was discarded, and 25 µL of neat supernatant was used for the analysis. The analysis was performed with MILLIPLEX® Bovine Cytokine/Chemokine Magnetic Bead Panel 1—Immunology Multiplex Assay kit (MilliporeSigma, Burlington, MA, USA), allowing detection of 15 analytes: IFN-γ, IL-1α, IL-1β, IL-4, IL-6, IL-8, IL-10, IL-17A, MIP-1α, IL-36RA, IP-10, MCP-1, MIP-1β, TNF-α, VEGF-A. Reading of the assay was performed with Bio-Plex 200 (Bio-RAD Laboratories, Hercules, CA, USA) and raw data were analyzed using Bio-Plex Manager software. The concentrations of the detected cytokines were calculated based on the fluorescence signal and a standard curve of known concentration standards according to the manufacturer’s instructions. Statistical analysis was performed in R. Initial screening on 78 samples was performed to assess the detectability of cytokines in milk. An additional 78 samples were analyzed for IL-4, IL-10, IFN-γ and TNF-α based on quality of standard curves, established roles in cellular immune responses, and known interactions with one another. Samples for this analysis were selected to maximize the representation of pathogen groups, covering 11 out of 13 groups, with at least three samples included for each group.

### Proteomic analysis

#### Sample preparation for liquid chromatography with tandem mass spectrometry (LC–MS/MS)

Immediately after aliquoting, the cell pellet for proteomic analysis was prepared from 40 mL of milk. Samples were centrifuged at 8000 *g* for 10 min at 4 °C, the supernatant and fat layer were discarded. Obtained cell pellets were washed by suspending in 10 mL of 2% citrate water (w/v) and centrifuged as before. The washed pellets were frozen at −20 °C and then transferred to −80 °C until the time of the analysis. Prior to analysis, thawed pellets were lysed with 200 µL of lysis buffer (50 mM Tris–HCl, pH 7.5, 4% SDS, 10 mM DTT) and bead beating with 0.2 g of 106 μm acid-washed beads (Sigma) using FastPrep-24 (MP Biomedicals). The protein extract was adjusted to a concentration of 1 mg/mL in lysis buffer, and 30 µL of this extract was used for sample preparation for LC–MS/MS with a gel-free suspension trapping method [25]. The proteins were first denatured at 95 °C for 10 min, followed by alkylation with 50 mM of iodoacetamide for 20 min in the dark at room temperature. Samples were acidified with 1/10 volume of phosphoric acid (PA) and loaded onto suspension trapping columns, built in 200µL EppendorfTM epT.I.P.S.TM as described [22, 25]. The columns consisted of 2 layers of C18 (Empore) topped with 12 layers of quartz filter (Munktell MK360). Samples were loaded onto columns in suspension trapping (STrap) buffer (90% methanol and 100 mM Tris–HCl at pH 7.1) by centrifugation at 4000 × *g* for 15 min. Proteins trapped in layers of quartz were washed with STrap buffer and 50 mM ammonium bicarbonate solution and digested with trypsin (0.45 µg of enzyme per 30 µg of proteins) in 50 mM ammonium bicarbonate for 1 h at 47 °C. Post digestion washes with 0.5% and 0.1% trifluoroacetic acid (TFA) transferred the digested peptides from quartz to C18 part of the column. Peptides were eluted with elution buffer: 80% acetonitrile (ACN) with 0.1% TFA. Eluted peptides were dried with a vacuum concentrator, resuspended in loading solution (2% ACN with 0.05% TFA) and sonicated in a water bath for 10 min prior to LC–MS/MS. The concentration of the peptide suspension was adjusted to 0.2 mg/mL and samples were analyzed by nano UPLC coupled to a trapped ion mobility spectrometry/quadrupole time of flight mass spectrometer (timsTOF- Pro, Bruker) for 60 min. Due to high complexity and labor-intensive nature of the sample preparation a subset of 56 samples was selected, for this analysis, in a way to maximize pathogen group representation, covering ten pathogen groups.

#### Mass spectrometry data analysis

Raw data from LC–MS/MS was analyzed in MaxQuant 2.6.6.0 software using label-free quantification (LFQ) algorithm with Andromeda search engine [26, 27]. Database searches were performed against the *Bos taurus* proteome (UniProt: UP000009136). Default parameters were applied, except for protein group identification, where the following settings were used: minimum peptides per protein group = 2 (with at least one unique peptide), minimum unique peptides per protein = 1, and at least one unmodified peptide with a minimum score of 1. Output file: proteinGroups resulting from MaxQuant search was used for further analysis, which was performed in R using DEP package [28]. Results were filtered, and contaminants were removed (Reverse, Only identified by site, and Potential contaminants). The mass spectrometry proteomics raw data files have been deposited to the ProteomeXchange Consortium via the PRIDE partner repository with the dataset identifier PXD069681.

#### Weighted gene co-expression network analysis (WGCNA)

Weighted correlation network analysis was performed on the proteomics data obtaned after normalization by variance stabilizing transformation in DEP package. Only protein groups present in more than 50% of the samples were included in the analysis. The WGCNA package was used to identify protein networks and co-expressed proteins using a soft threshold power of β = 12 and a dynamic tree-cutting algorithm with the following parameters: minimum module size of 20, deepSplit set to 2, and a merge cut height of 0.2 [29]. A hybrid signed adjacency matrix was applied. Module membership (kME) was assessed by calculating the Pearson correlation between individual proteins and module eigenprotein as described before [22, 30]. To investigate patterns of protein expression that could potentially be related to inflammation and infection, the correlation between module eigenvectors and SCC was investigated.

#### Pathway enrichment analysis

Functional annotation of proteome database (performed with eggNOG version 5.0.2) was merged with WGCNA analysis results to perform GO enrichment analysis, KEGG pathway enrichment, and gene ontology analysis of proteins in each module using clusterProfiler R package [31]. Proteins within each module were analyzed for differential expression across pathogen groups using R and the Tidyverse package [32]. The significance of observed differences was evaluated with the Kruskal–Wallis test.

## Results

### Presence of pathogen in microbiota data

Grouping samples into pathogen groups, based on the pathogens detected in the milk microbiota, resulted in 13 groups (Table 2). In 143 samples (42% of the samples), no presence of the dominating bacteria was detected, with the percentage of reads exceeding 30%. The proportion of negative quarters was 47% before drying off, increased to 61.2% at the beginning of the next lactation, and then declined to 41.8% by the end of lactation. NASM, which included the group of samples positive for *S. chromogenes, S. epidermidis,* and “other NASM”, was the most abundant pathogen group, accounting for 13.4% of total samples, followed by *C. bovis* 6.8%, *Enterococcus faecalis* (*E. faecalis*) 4.2%, and *S. uberis* 3.9%.

**Table 2:** **IBC and abundance (%) of defining bacterial taxa in samples across lactation period in different pathogen groups**

Pathogen group name	Number of samples	Average (± sd) of log_10_ of Bacterial counts (IBC/mL)	% of quarter samples classified into the group and stratified by the lactation period				Defining taxa
			Before dry period	Beginning	Middle	End	
Negative	143	3.5 (± ± 0.67)	47.4	61.2	48.6	41.8	Negative
Minor pathogens	11	3.1 (± 0.25)	5.3	0	6.8	4.5	*Kocuria* spp./*L. cremoris or lactis*/*Romboutsia timonensis*/*Sphingomonas suaedae*
*Aerococcus*	11	3.37 (± 0.55)	3.5	4.7	2.7	4.5	*A. urinaeequi*/*A. viridans*
*Enterococcus*	14	4.1 (± 0.55)	3.5	3.5	5.4	7.5	*E. faecalis*
*C. bovis*	23	3.53 (± 0.47)	12.3	10.6	6.8	3	*C. bovis*
Other *Corynebacterium*	8	3.73 (± 0.13)	1.8	2.4	2.7	4.5	*C. camporealensis*/*C. parakroppenstedtii*/*C. xerosis*/*C.* spp.
*S. aureus*	9	5.01 (± 1.27)	1.8	3.5	4.1	3	*S. aureus*
*S. chromogenes*	13	3.54 (± 0.53)	5.3	7.1	1.4	4.5	*S. chromogenes*
*S. epidermidis*	14	3.48 (± 0.37)	3.5	2.4	10.8	3	*S. epidermidis*
Other NASM	18	3.54 (± 0.52)	1.8	2.4	6.8	14.9	*S. xylosus*/*S. haemolyticus/S. Hominis*/*S.* spp.
*S. dysgalactiae*	2	4.81 (± 0.25)	1.8	1.2	0	0	*S. dysgalactiae*
*S. uberis*	13	3.86 (± 0.27)	8.8	1.2	2.7	7.5	*S. uberis*
Other *Streptococcus*	4	5.87 (± 1.67)	3.5	0	1.4	1.5	*Streptococcus.* spp.

### Bacterial and somatic cell counts and cellular subsets

**Figure 2:**
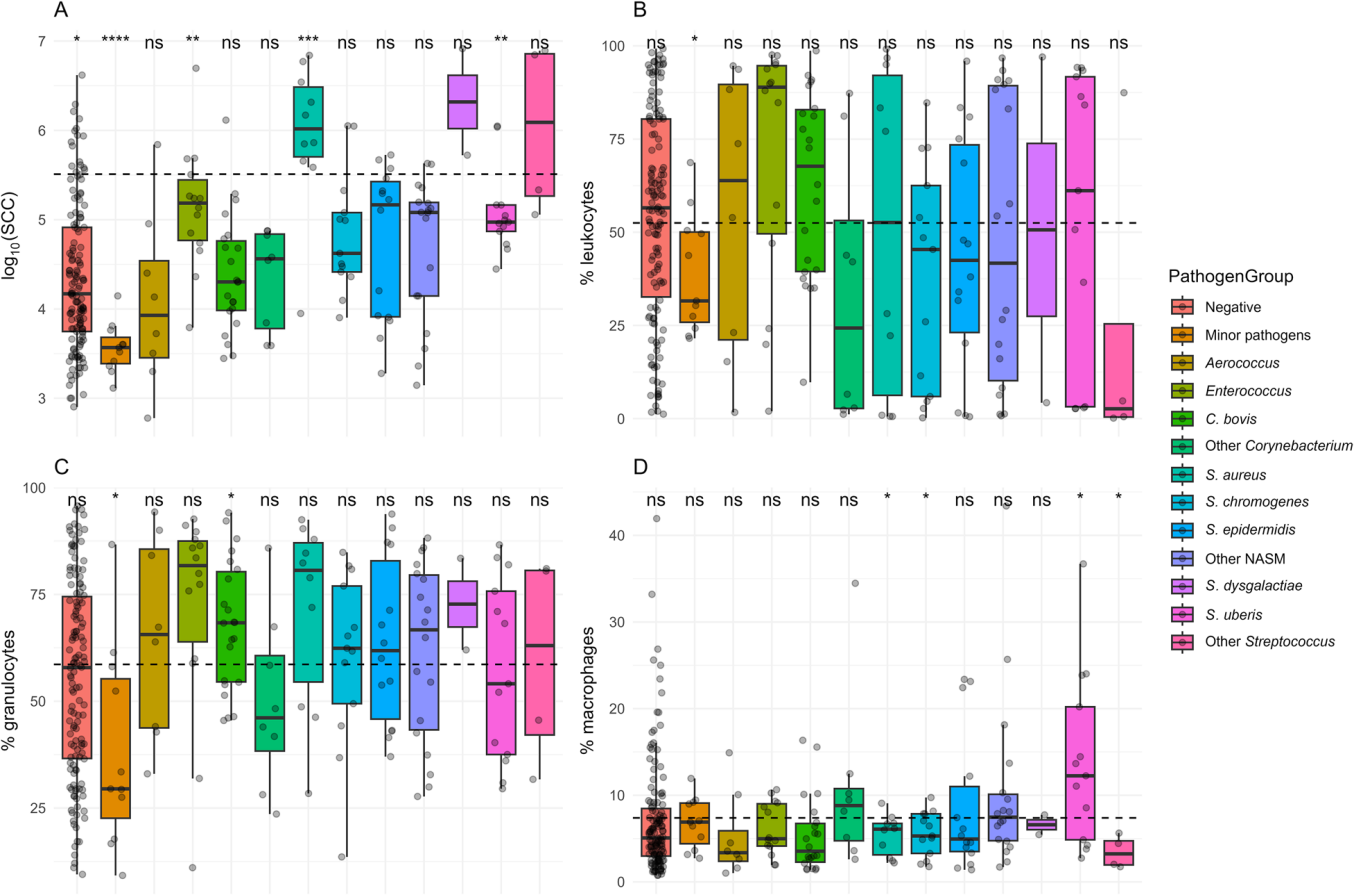
**General overview of the SCC (A), Leucocytes (B), Granulocytes (C), and Monocytes + macrophages (D) differentiation based on the pathogen groups**. A significant difference marked with *(0.01 < *p* ≤ 0.05), **(0.001 < *p* ≤ 0.01), ***(0.0001 < *p* ≤ 0.001), ****(*p* ≤ 0.0001), ns—no significant difference (*p* > 0.05), compared to the global mean of all samples.

The bacterial counts (log_10_ IBC/mL) showed the highest values for samples from groups Other *Streptococcus* and *S. aureus* groups with values above 5 (log_10_ IBC/mL), and S*. dysgalactiae* and *Enterococcus* groups with values above 4 (log_10_ IBC/mL) (Table 2). The lowest values of IBC/mL were present in samples from groups Minor pathogens and *Aerococcus* groups. When the level of SCC in each sample was grouped by the presence of pathogen, the two groups Other *Streptococcus* and *S. aureus* had the highest level of SCC compared to the mean of all the samples (Figure 2A). The group *S. dysgalactiae* presented a higher level of SCC while a significantly lower level of SCC was observed for samples from the Minor pathogens, Negative and *S. uberis* groups.

The somatic cells were further characterized by flow cytometry into relative numbers of leucocytes, granulocytes, and monocytes + macrophages (defined as CD14 + cells). The *S. aureus* and Other NASM groups showed the highest percentage distribution of leucocytes, whereas the highest values for these cells fraction overall were present in the *Enterococcus* group. One of the lowest percentage of leucocytes was detected in Other *Streptococcus* group (Figure 2B). The highest mean granulocytes percentage was observed in samples from *Enterococcus* and *S. aureus* groups, with Minor pathogens group having significantly lower values (Figure 2C). The groups Other *Streptococcus*, *S, aureus* and *S. chromogenes* had significantly lower percentage of monocytes + macrophages whereas *S. uberis* group had significantly higher values (Figure 2D). Significance was measured for each pathogen group vs mean of all samples with T-test.

### Cytokines

**Figure 3:**
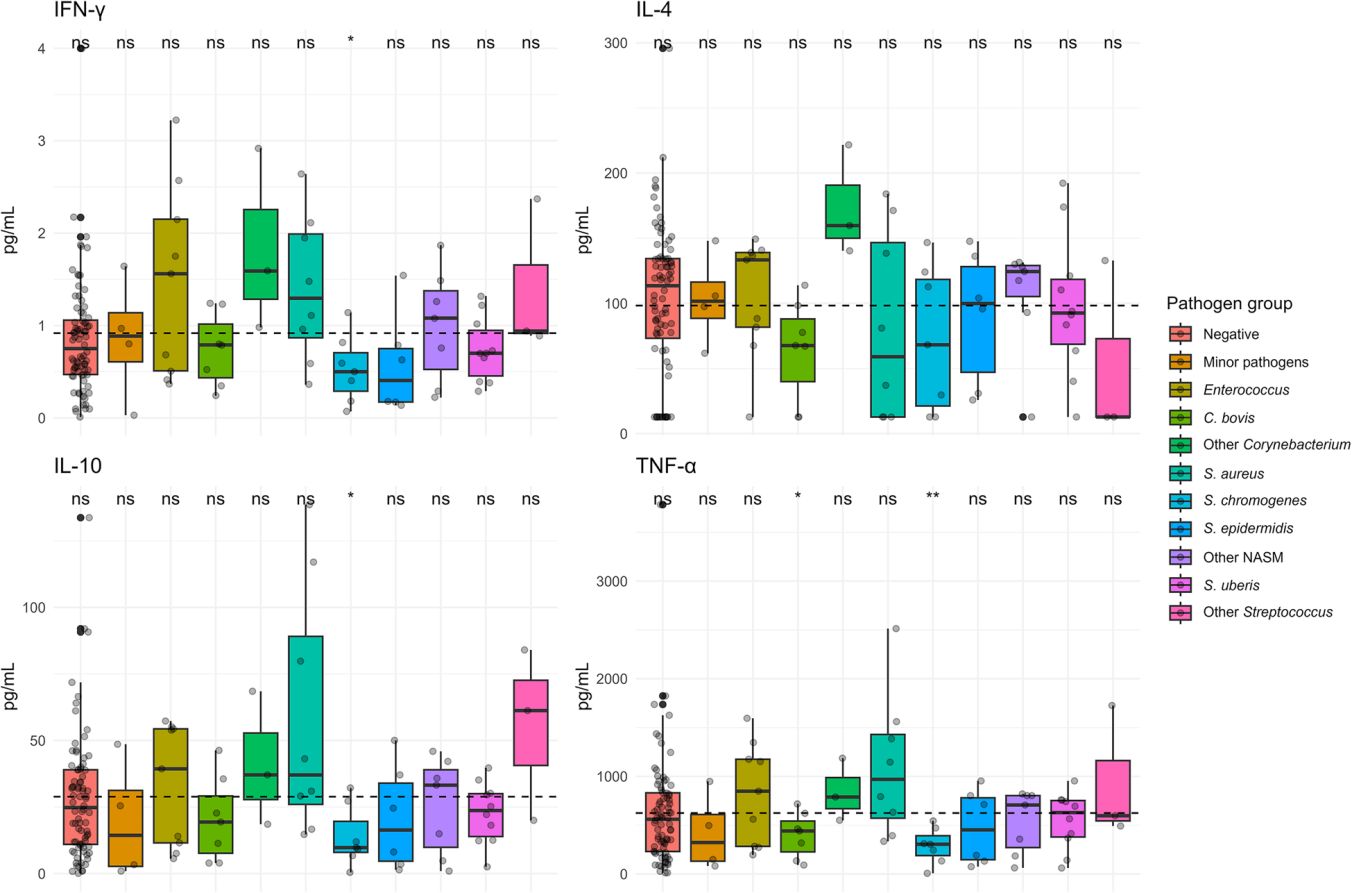
**Calculated cytokines concentrations in samples across different pathogen groups.** Dotted line indicating mean value for each analyte. A significant difference marked with *(0.01 < *p* ≤ 0.05), **(0.001 < *p* ≤ 0.01), ns—no significant difference (*p* > 0.05), compared to the global mean of all samples.

Cytokines analysis with multiplexing ELISA of samples grouped by pathogen group showed significantly lower levels of IFN-γ, IL-10 and TNF-α in samples classified within the *S. chromogenes* group. *C. bovis* group samples also had significantly lower levels of TNF-α (Figure 3). Samples from *S*. *aureus* group had higher levels of IFN-γ, IL-10, and TNF-α and lower levels of IL-4 compared to the global mean. These samples also showed the greatest distribution of values for IL-4 and IL-10 compared to the other groups. Similar patterns to *S. aureus* group were presented in samples from the Other *Streptococcus* group. For all cytokines examined, the *S. uberis,* Minor pathogens, and Negative groups exhibited mean values closely aligned with the overall mean. The level of all the tested cytokines was higher for the *Enterococcus* group compared to the overall mean. Like *Enterococcus*, the Other *Corynebacterium* group showed a high level of cytokines, with one of the highest values for IL-4. However, *C. bovis* group had lower levels of all the cytokines than the overall mean (Figure 3).

**Figure 4:**
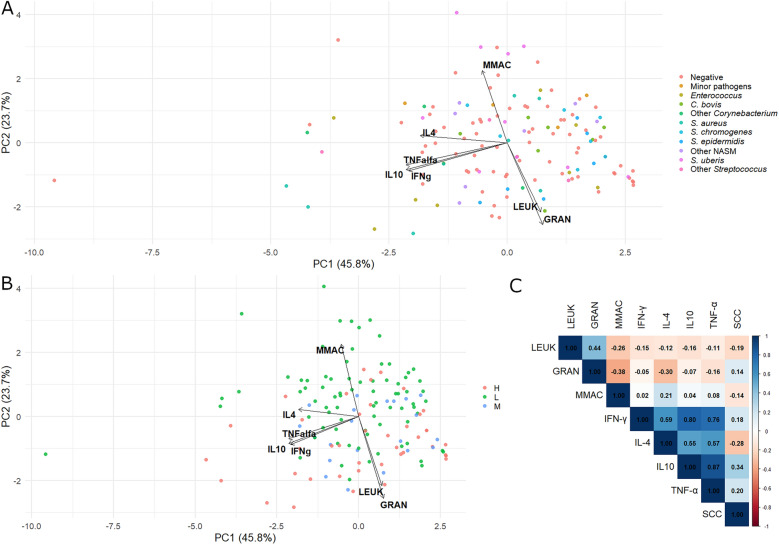
**PCA of cytokines and somatic cells colored by Pathogen group (A) and SCC value (B), correlation of cytokines and somatic cells (C)**. LEUK—leukocytes, GRAN- granulocytes, MMAC—monocytes + macrophages.

Principal component analysis of cytokines and somatic cells population showed a positive association between total leukocyte counts (LEUK) and granulocytes (GRAN) (Figure 4) and samples with high SCC values tended to align with those variables (Figure 4A). However, there was no association of any of the variables with pathogen group (Figure 4B). Correlation analysis of the same variables and overall SCC level showed positive correlation of SCC with IL-10 and negative with IL-4, as well as negative correlation of IL-4 with granulocytes (Figure 4C).

### Host proteomic analysis

Proteomic analysis included total of 56 quarter samples, therefore not all pathogen groups were represented in this data subset. The proteomic analysis detected a total number of 3570 proteins. The samples analyzed by proteomics contained samples from 10 different pathogen groups: Negative, *C. bovis*, *Enterococcus*, *S. aureus*, *S. chromogenes*, *S. epidermidis*, Other NASM, *S. dysgalactiae*, *S. uberis* and Other *Streptococcus*, with the biggest group of the samples classified as group Negative (36%). Weighted gene expression network analysis grouped the 1352 proteins found in more than 50% of the samples into modules using correlation of expression between each pair of two proteins and using hierarchical clustering of topology overlap measure to define the modules and module membership of the proteins [29, 30]. The analysis identified 10 modules of proteins with similar expression patterns, with an average of 129 proteins per module, and the smallest consisting of 45 (module 3) and the largest 338 (module 6) (Additional file 2).

**Figure 5:**
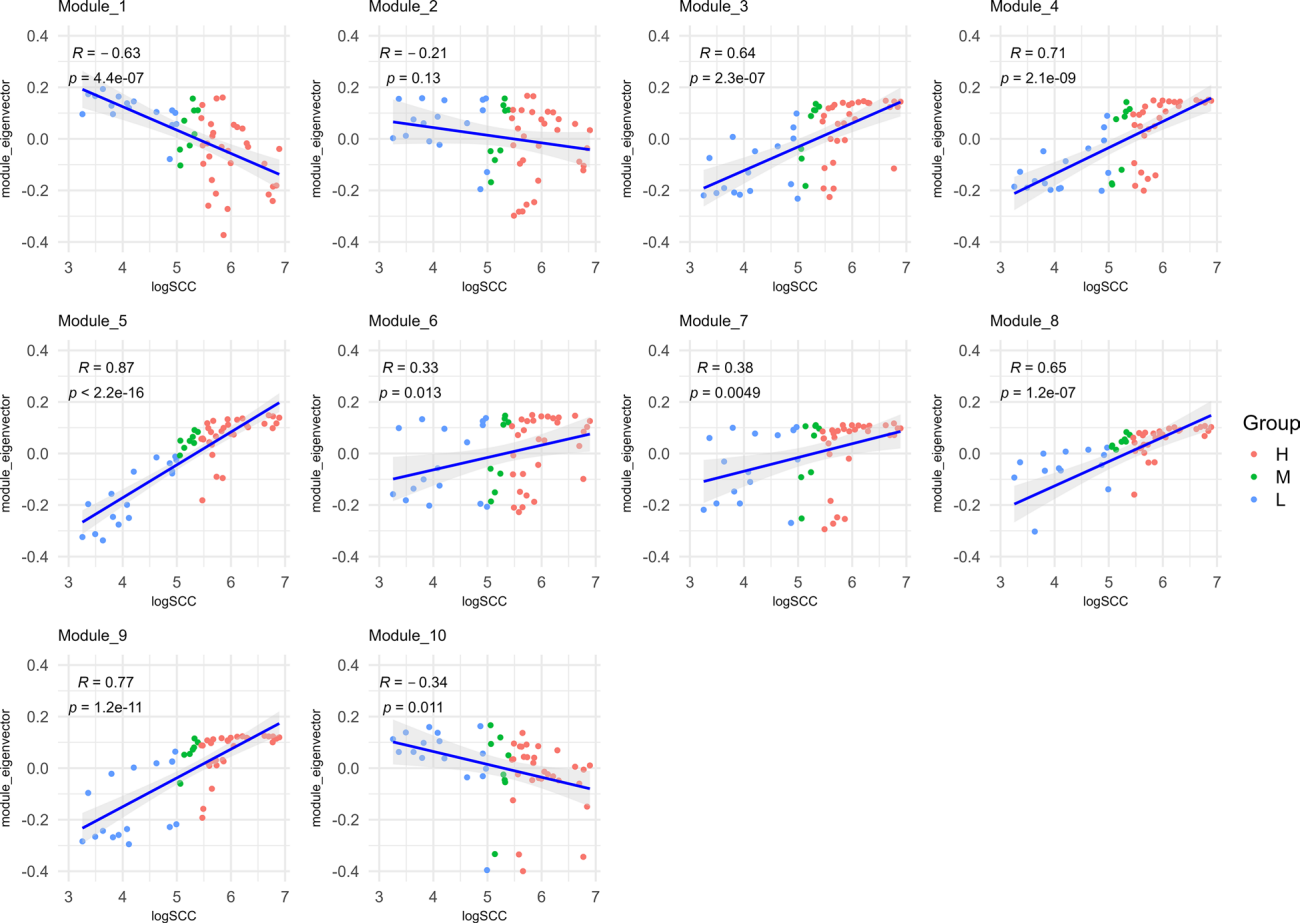
**Correlation of SCC with the module eigenvector across 10 modules.** H-high, M-medium, L-low SCC.

Correlation analysis of module eigenvectors and SCC analysis showed that two modules of proteins (modules 1 and 10) had a significant negative correlation with the increase in SCC (Figure 5), while seven modules had a significant positive correlation, with module 5 having the strongest correlation (R = 0.87). Module 2 showed protein expression independent of the SCC level, while Modules 6 and 7 had the lowest correlation with SCC levels (Figure 5). Expression level in modules was also dependent on pathogen group, modules 4, 5 and 8 showed expression independent of pathogen group whereas the rest of the modules presented some level of variablity (Additional file 3).

Modules 1, 2, 10, 5, 6 and 7 were further investigated by functional annotation of the proteins assigned to these modules. This was done by investigating gene ontology and enriched pathways in each of the modules. KEGG pathway enrichment analysis of module 1 showed that the module had significantly enriched pathways of lipid metabolism, particularly fatty acid biosynthesis and steroid biosynthesis (Fold enrichment 41 and 37, respectively, and *p*.adjust 0.003). Folding, sorting, and degradation of genetic material: SNARE interactions in vesicular transport were also enriched in the same module (Fold enrichment 22, *p*.adjust 0.005) (Additional file 4).

Module 2 was highly enriched in proteins from pathways of response to bacterial infections as well as cell growth and death, particularly iron-dependent ferroptosis (fold enrichment 7 and 6, respectively). Modules 6 and 7, which had weak positive correlation with SCC levels, were enriched in proteins involved in neurodegenerative disorders like Hungtington disease, Alzheimer disease or Parkinson disease. Deeper investigation of those pathways showed that present genes were responsible for mitochondrial functions as well as protein processing in the cells. Besides that, both of the modules were highly enriched in proteins for the Citrate cycle (TCA), respectively 11- and 15-fold enrichment (*p*.adjust 1.33e-05 and 3.0e-4). Module 5 was enriched with multiple pathways of the immune system and immune response, as well as pathways of basic biosynthesis and metabolism. The most enriched pathway in this module was Neutrophil extracellular trap formation (Fold enrichement 10, *p*.adjust 2.538e-07). Other highly enriched pathways in module 5 were the Fcγ receptor-mediated phagocytosis and leucocyte transendothelial migration (Fold enrichment 8 and 12). Module 10 did not present any enriched pathways, probably due to the low number of member proteins in the module.

A total of 67 proteins were significantly differently expressed across modules 1, 2, 6, 7, and 10, depending on the pathogen group; the rest of the modules did not exhibit differential protein expression based on the pathogen group (Additional file 5). Out the 67 proteins, 19 were associated with immune system functions (Table 3, Additional file 6). Proteins of a particular interest due to their role in inflamation and mastitis were lactoferrin (lactotransferrin), JAK1 and TLR2.

**Table 3:** **Significantly differently expressed proteins depending on pathogen group across the modules—chosen proteins involved in immune response (full table in Additional file 5)**

Protein	Gene	Name	Function/process/localization	*p*-value
A0A0P0QLR2	*TLR2*	Toll-like receptor 2	Innate immune response, regulation of inflammatory response	0.022
G3N2D8	*GGT1*	Glutathione hydrolase	Regulation of immune system process, regulation of inflammatory response	0.027
P18892	*BTN1A1*	Butyrophilin subfamily 1 member A1	Regulation of cytokine production, T-cell receptor signaling pathway, milk-fat droplet secretion	0.017
P15396	*ENPP3*	Ectonucleotide pyrophosphatase	Basophil activation, negative regulation of inflammatory response, negative regulation of mast cell activation and proliferation	0.014
Q6QRN7	*TMBIM1*	PP1201 protein	Negative regulation of apoptosis pathways	0.017
Q8MI01	*MUC15*	Mucin-15	Apical surface of mammary epithelial cells	0.0186
Q32LK2	*CYSTM1*	Cysteine-rich and transmembrane domain-containing protein 1	Neutrophil degranulation	0.024
A5HLY3	*LTF*	Lactotransferrin	Antibacterial humoral response	0.030
A0A4W2HT32	*LRRC8A*	Volume-regulated anion channel subunit LRRC8A	Pre-B-cell differentiation	0.020
A0A4W2H2R9	*ALOX15*	Polyunsaturated fatty acid lipoxygenase ALOX15	Negative regulation of adaptive immune response	0.008
A0AAF6YJV2	*GNAI1*	Guanine nucleotide-binding protein G(i) subunit alpha-1	T-cell migration	0.048
A0A4W2GBE1	*VPS37B*	VPS37B subunit of ESCRT-I	Positive regulation of viral budding form host cells	0.021
A0A3Q1LQY1	*CNTFR*	Ciliary neurotrophic factor receptor subunit alpha	Type 1 cytokine receptor family	0.040
A0A4W2HPE5	*LOC113888430*	Multidrug resistance-associated protein 4	Apical cell membrane homeostasis, migration of dendritic cells	0.040
A0A4W2IMF4	*ARL6IP5*	PRA1 family protein	Positive regulation of intrinsic apoptotic pathway in response to oxidative stress	0.033
F1N0D6	*JAK1*	Tyrosine-protein kinase	Intracellular signal transduction, IFN signaling	0.033
F1N261	*LYN*	Tyrosine-protein kinase	B-cell homeostasis, B-cell receptor signaling pathway, dendritic cells differentiation, inflammatory response regulation	0.047
O46631	*SIRPA*	Tyrosine-protein phosphatase non-receptor type substrate 1	Positive regulation of phagocytosis, positive regulation of T-cell activation	0.014
A0A4W2CJB3	*FGG*	Fibrinogen gamma chain	Negative regulation of apoptotic process	0.041

*p*-value: Kruskal–Wallis test for differential protein expression in the pathogen group.

## Discussion

Our study employed a non-invasive, multimethod approach to investigate the bovine immune response to the presence of pathogens in the udder. Our findings demonstrated different patterns of SCC, cell populations and cytokines in the milk associated with the presence of bacteria such as *S. chromogenes* and other species of *Corynebacteria,* including *C. bovis*. Additionally, we analyzed the host proteome to identify patterns of co-expressed proteins and examined their correlation with the SCC levels and the specific pathogen present.

The somatic cell counts analysis revealed the association of high SCC levels with the *S. aureus* group and a lower level when NASM (S*. epidermidis*, *S. chromogenes,* and Other NASM groups) were present, as already noted by other studies [18, 33]. Cytokine analysis showed high levels of TNF-α and IFN-γ as well as low levels of IL-4 associated with the *S. aureus* group*,* which has been previously shown [34, 35]. Elevated levels of TNF-α and IFN-γ suggest an intensified proinflammatory immune response. TNF-α, which can be produced by a wide variety of somatic cells but mostly monocytes or macrophages, increases upon stimulation by components of the bacterial cell wall and in response to other cytokines, like IFN-γ [36, 37]. In our case, samples from *S. aureus* group were characterized by the highest IBC levels among all groups, likely explaining the increased production of TNF-α. It has also been suggested that an increase in TNF-α related to the presence *of S. aureus* can stimulate production of anti-inflammatory IL-10, which we also observed at high levels in samples for the *S. aureus* group [36, 38]. IFN-γ, produced by T-lymphocytes and natural killer cells, induces phagocytotic activity of monocytes, macrophages and neutrophils. It was suggested to be linked with persistent infection with high IBC and was detected to be elevated upon *E. coli* and *S. aureus* infection [36, 39].

In contrast to *S. aureus*, samples from the *S. chromogenes* and *S. epidermidis* groups exhibited low SCC and an inverse cytokine expression profile, suggesting a limited or absent interaction between these species and the host immune system. Piccart et al. [12] reported that the host response to *S. chromogenes* may vary depending on the strain’s origin, which could be relevant in the present context. Unfortunately, we did not perform analysis at the strain level, but we suspect that NASM strains present in our study belong to the group that has little to no interaction with the host response or were present at a level that did not trigger the immune response.

The Negative group samples displayed a wide range of SCC levels. This variation might be attributed to periods of lactation, as our study collected samples through the full lactation cycle, and the level of samples regarded as negative was higher at the beginning of lactation than at any other stage. At the same time, SCC is known to be higher shortly after calving at the beginning of lactation [33]. However, we cannot exclude that the Negative group also contained samples post-infection, especially in later stages of lactation. Potentially, some of those samples presenting patterns of inflammation can be attributed to past infection with no longer detectable levels of the pathogen, such as a long-lasting inflammatory status after infection with minor pathogens or no prior infection at all [40]. Samples from groups like *Enterococcus* and *C. bovis* had below-average SCC but displayed somatic cell distribution typically associated with inflammation. These higher levels of leukocytes, with an average above 80%, suggest an intensified immune response despite low average SCC [33]. Those groups also showed higher levels of granulocytes, suggesting an active immune response to the presence of these pathogens. Low levels of IL-4 detected in samples from *C. bovis* group can be related to an ongoing inflammation as low levels of this cytokine in milk are typically detected in mastitis caused by various pathogens even if we have not found previous reports associating IL-4 with *C. bovis* [34, 35, 41, 42].

Interestingly, samples classified to the Minor pathogens group had the lowest SCC of all samples; this group consisted of taxa quite commonly found in milk but rarely associated with the disease, like *Kocuria* spp. and considered health-promoting like *Lactcoccus cremoris* [43, 44]. This group was also characterized by low levels of TNF-α, IFN-γ, and IL-10, suggesting a lack of specific interaction between these species and the host immune system. In contrast, samples from the Other *Corynebacterium* group showed particularly high levels of IL-4 alongside low SCC levels. This pattern could indicate a protective role for these species, consistent with the anti-inflammatory properties of IL-4 [41]. Alternatively, a high level of IL-4 combined with a high level of TNF-α may reflect the late stage of an infection or a transition toward resolution, as TNF-α is typically associated with active immune responses [45]. Nevertheless, the combination of non-elevated SCC with a relatively low IBC and with the presence of typically nonpathogenic *Corynebacterium* species might suggest a potential protective role of this bacterial group.

Proteomic analysis of host response is used to determine or test for marker proteins of IMI; however, there is a limited number of studies that focus on the entire proteome. Shotgun proteomics has been previously used to study the host proteome in mastitis caused by *S. aureus* and *S. uberis* by Winther et al. [22]. In this study, we covered those and an additional 8 more pathogen groups with varying SCC levels. We applied co-expression network analysis to identify modules of proteins that are similarly co-expressed and to investigate these further. The module eigenvector correlated with SCC levels and pathway enrichment analysis revealed associations with immune response and inflammatory pathways.

Particularly highly enriched pathway in module 5, positively correlated with SCC level, was the Neutrophil extracellular trap (NETs) formation. NETs has been shown to have higher expression levels related to *S. aureus* presence and be related to inflammatory status in cows, sheep, and goats [22, 46–48]. However, in our data there is no pathogen-dependent difference in this protein expression. NETs formation from neutrophils is typically triggered by the presence of pathogens, and *S. aureus* is known for its ability to induce NETs formation and have systems to evade their activity [49]. Particularly the NET formation without neutrophil death can be induced by *S. aureus* virulence factors, such as Panton-Valentine leucocidin or protein A. Protein A is also used by *S. aureus* to evade immune system by binding to the Fcγ receptor, which can prevent bacterial cell opsonization and phagocytosis [50]. In our data, both the NET formation pathway and the Fcγ receptor-mediated phagocytosis had the same expression pattern and were both upregulated in module 5, which positively correlated with SCC levels. Fcγ receptor was also upregulated in module 4, which is also positively correlated with SCC levels. Interestingly, the NETs and the Fcγ receptor pathway enriched modules, didn’t show significant difference in protein expression based on pathogen group. Therefore, it appears that these proteins are part of the general inflammatory response and are not pathogen-dependent.

Lactoferrin (also known as lactotransferrin) showed significantly different expression depending on pathogen group in module 2. Lactoferrin, an iron-binding protein, is a key element of the first line of defense of the immune system, as it possesses antimicrobial and anti-inflammatory properties. It is naturally present in various body sites, such as milk, saliva, and mucosal membranes. Lactoferrin presence in milk varies depending on the stage of lactation, and it has been shown to correlate with SCC levels and typically increases with the infection [51, 52]. Lactoferrin has been reported to be an endogenous regulatory system that modulates NETs formation and activity carried by neutrophils, thereby decreasing the intensity of the immune response. It can bind to DNA, inhibit NETosis, limit NETs release, and reduce neutrophil-mediated immune response [53]. In our study, we found that NETs formation proteins and lactoferrin were classified in different modules and did not seem to have been enriched in the same samples, meaning a different expression pattern in the bovine proteome. Lactoferrin expression is strongly dependent on pathogen group, with the highest level being in the *Enterococcus* group. It has been reported that this protein plays a role in antimicrobial defence, particularly against enteric infections. Lactoferrin sequesters iron from the environment, preventing bacteria from scavenging it, but it can also bind to lipid A of LPS and disrupt the membrane of Gram-negative bacteria [54]. It also plays a role in regulating immune response particularly can reduce production of cytokines which expression is stimulated by LPS such as TNF-α or IL-6 [55]. This could explain why TNF-α levels in samples from the *Enterococcus* group were lower than in the *S. aureus* group, which exhibited much lower levels of lactoferrin. High levels of lactoferrin were also observed in pathogen groups of *S. dysgalactiae, S. uberis* and in lower level in the Other *Streptococcus* group. A high expression in subclinical mastitis caused by species of *Streptococcus* was noticed before by Chaneton and coauthors [56] with slightly lower expression related to *Staphylococcus*, similar to our observations. It was also suggested that the *S. uberis* is resistant to lactoferrin and uses lactoferrin to increase adherence to epithelial cells of the mammary gland [56, 57].

In our dataset we detected another protein showing pathogen-dependent expression. JAK1 is a key component of the JAK-STAT signaling pathway. JAK1 is involved in the activation and signaling of interferons, including IFN-γ, and plays a crucial role in driving the production of various proinflammatory cytokines, such as IL-6, TNF-α, and IL-1β [58]. Interestingly, in this study, samples from the *C. bovis* group, which exhibited the highest JAK1 expression, did not show elevated levels of IFN-γ or TNF-α; rather, these cytokines were observed at relatively low levels. This discrepancy may reflect the fact that cytokine expression is regulated by multiple signaling pathways in addition to JAK-STAT, like activation through Toll-like receptors (TLR) [59]. The absence of other key JAK-STAT components in our dataset further suggests that this pathway may not be playing a significant role in the cytokine activation in this study.

Protein A0A0P0QLR2, which was identified as Toll-like receptor 2 (TLR2), showed different expression levels depending on pathogen group. TLR2 has been previously associated with the presence of pathogens at different levels, particularly with *S. aureus*, having the highest influence on expression [60]. In our results, however, the expression of this protein was not markedly elevated in samples from the *S. aureus* group. Instead, the highest levels were observed in the *S. epidermidis* and Other NASM groups. TLR2 can recognise multiple PAMPs originating from different bacteria and it is associated with both inflammation and IMI-driven mastitis [61, 62]. Lakshmi and coauthors [63] investigated TLR2 expression in subclinical and clinical mastitis caused by *S. aureus* IMI and showed that expression was significantly higher in subclinical mastitis. TLR2 expression in clinical mastitis was almost the same as in the sample regarded as negative. In our results TLR2 expression varied besed on pathogen group but in *S. aureus* group had the widest range. So TLR2 could potentially be a candidate for a subclinical mastitis marker, though it needs further investigation to determine whether differences in expression levels could be consistently attributed to different pathogens and if the effect is appliable to all mastitis pathogens.

In this study, we used three different approaches for mapping immune system activity, all of which were noninvasive and used only hind milk collected via milking. The cytokines panel proved to be an effective and quick method for measuring inflammatory response, using a minimal volume of sample. The integrated approaches of flow cytometry and proteomic analysis of somatic cells demonstrated patterns of host immune response to different pathogens.

A methodological consideration of this work is the potential interdependence between udder quarters. Although such effects have been reported, especially in clinical mastitis caused by major pathogens, other studies have found no influence on SCC in healthy or mildly affected quarters. Our study did not include clinical mastitis and covered a broad range of bacterial species for which inter-quarter effects are largely unknown. Because our analyses focused on within-sample immune features and the dataset was unbalanced across cows, accounting for interdependence would not have altered interpretation. Assessing whether these effects apply to a wider range of pathogens would require a separate, targeted study.

Another limitation of this study is that not all analytical methods were applied to the full set of samples. Due to the time-consuming and labor-intensive nature of cytokines detection and proteomic workflows, these analyses were conducted on smaller subsets of samples. These subsets were selected to be as representative as possible of the full dataset, with the aim of including the greatest number of pathogen groups across all methods. However, the reduced sample sizes may have limited statistical power and the ability to detect smaller effects. To ensure transparency, the number of samples and pathogen groups included in each analysis is reported in the Methods section.

## Conclusions

Different bacterial species induced distinct immune responses in the bovine mammary gland. The observed variation in immune activation suggests that some pathogens trigger a stronger inflammatory reaction, whereas others may persist with comparatively limited host immune stimulation. The pathogen-related variability in TLR2 abundance in somatic cells suggests that it could serve as a candidate marker for pathogen-specific subclinical mastitis. These results demonstrate that immune activation in the mammary gland is pathogen-specific and identify candidate proteins that may contribute to distinguishing infections caused by different bacterial species, provided further investigation.

## Supplementary Information

**Additional file 1.** **Fluorecence minus one (FMO) controls.** Gating strategies for cell identification in flow cytometry - controls.

**Additional file 2.** **Modules eigenvectors.** Protein membership for eigenvectors in modules.

**Additional file 3.** **Protein expression in modules.** Protein expression level in modules.

**Additional file 4.** **KEGG analysis in modules**. KEGG enrichment analysis for each module.

**Additional file 5.** **Differently expressed proteins between pathogen group.** List of 67 differently expressed proteins.

**Additional file 6.** **Proteins involved in immune response with differential expression between pathogen groups.** Expression of 19 differently expressed proteins related to immune function.

## Data Availability

Proteomics data generated in this study have been deposited to the PRIDE partner repository of the ProteomeXchange Consortium—accession number: PXD069681. Data supporting the findings of this study, including processed results and analyses of the raw data, are provided within the manuscript and its additional files.
