# Supplementary material for: Bacteria-dependent modulation of immune responses in the bovine udder
Source: Vet Res. 2026 Apr 10;57:75. doi: 10.1186/s13567-026-01754-6 (PMC13181906; doi:10.1186/s13567-026-01754-6)
Supplement: Supplementary file 1 — Additional file 1. Fluorecence minus one (FMO) controls. Gating strategies for cell identification in flow cytometry - controls. [file 13567_2026_1754_MOESM1_ESM.pdf]

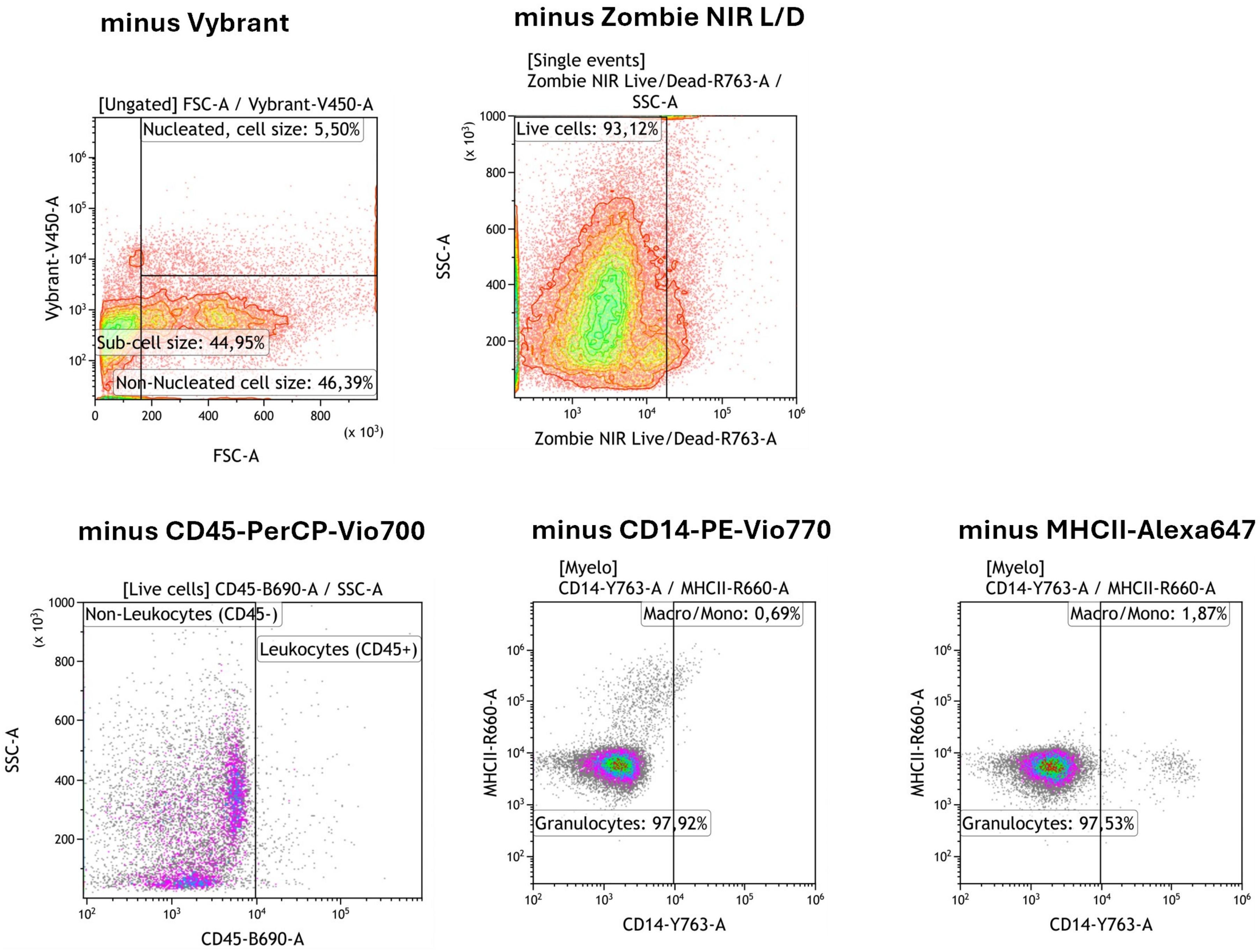

Supplementary figure 1. Fluorescence minus one controls shown for the markers used in flow cytometry, applying the gating strategy as shown in Figure 1. showing relevant plots and gates for the respective markers.
