# Supplementary figures and images for: Bacteria-dependent modulation of immune responses in the bovine udder
Source: Vet Res. 2026 Apr 10;57:75. doi: 10.1186/s13567-026-01754-6 (PMC13181906; doi:10.1186/s13567-026-01754-6)

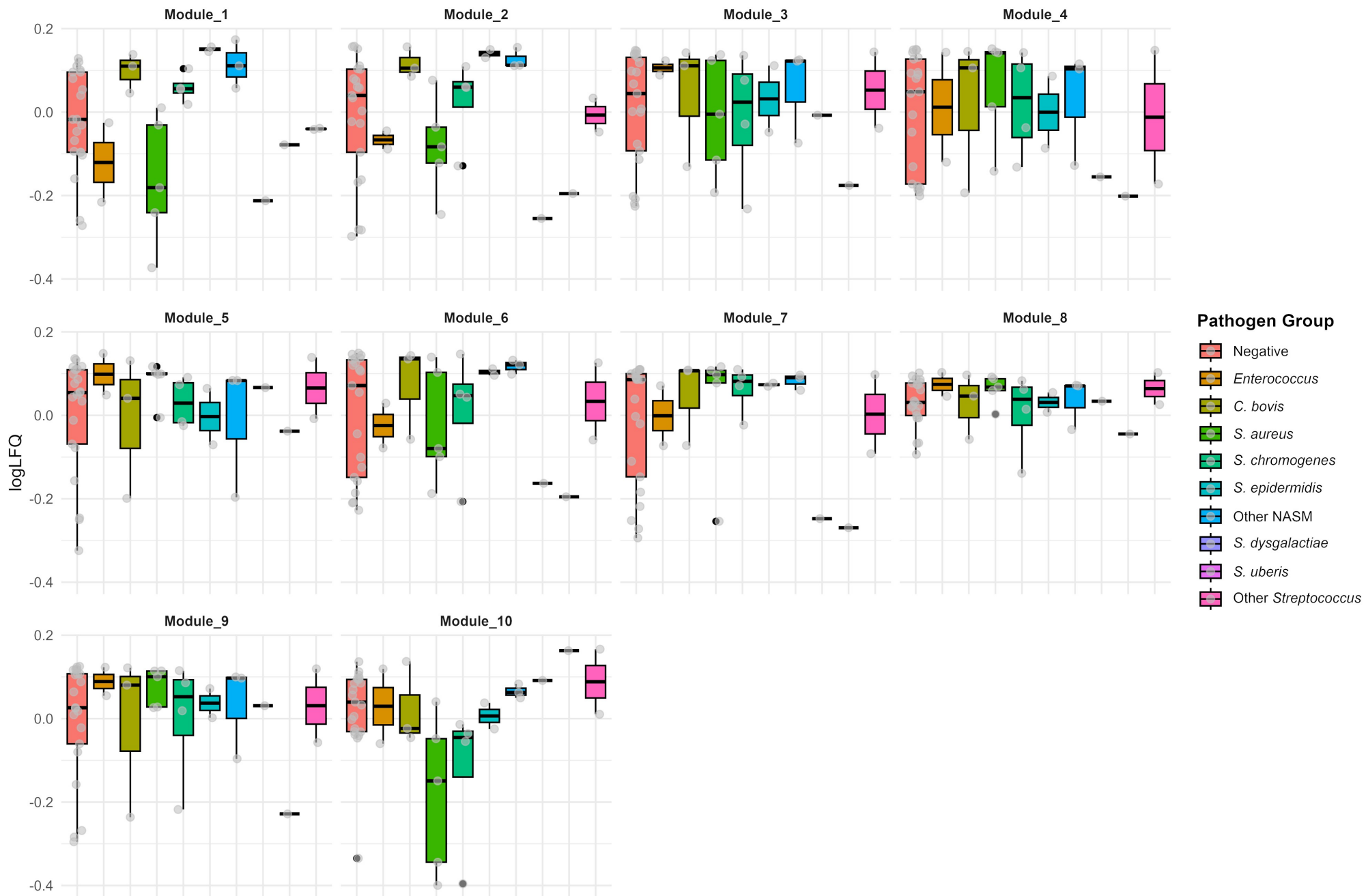

Supplement: Supplementary file 3 — Additional file 3. Protein expression in modules. Protein expression level in modules. [file 13567_2026_1754_MOESM3_ESM.pdf]

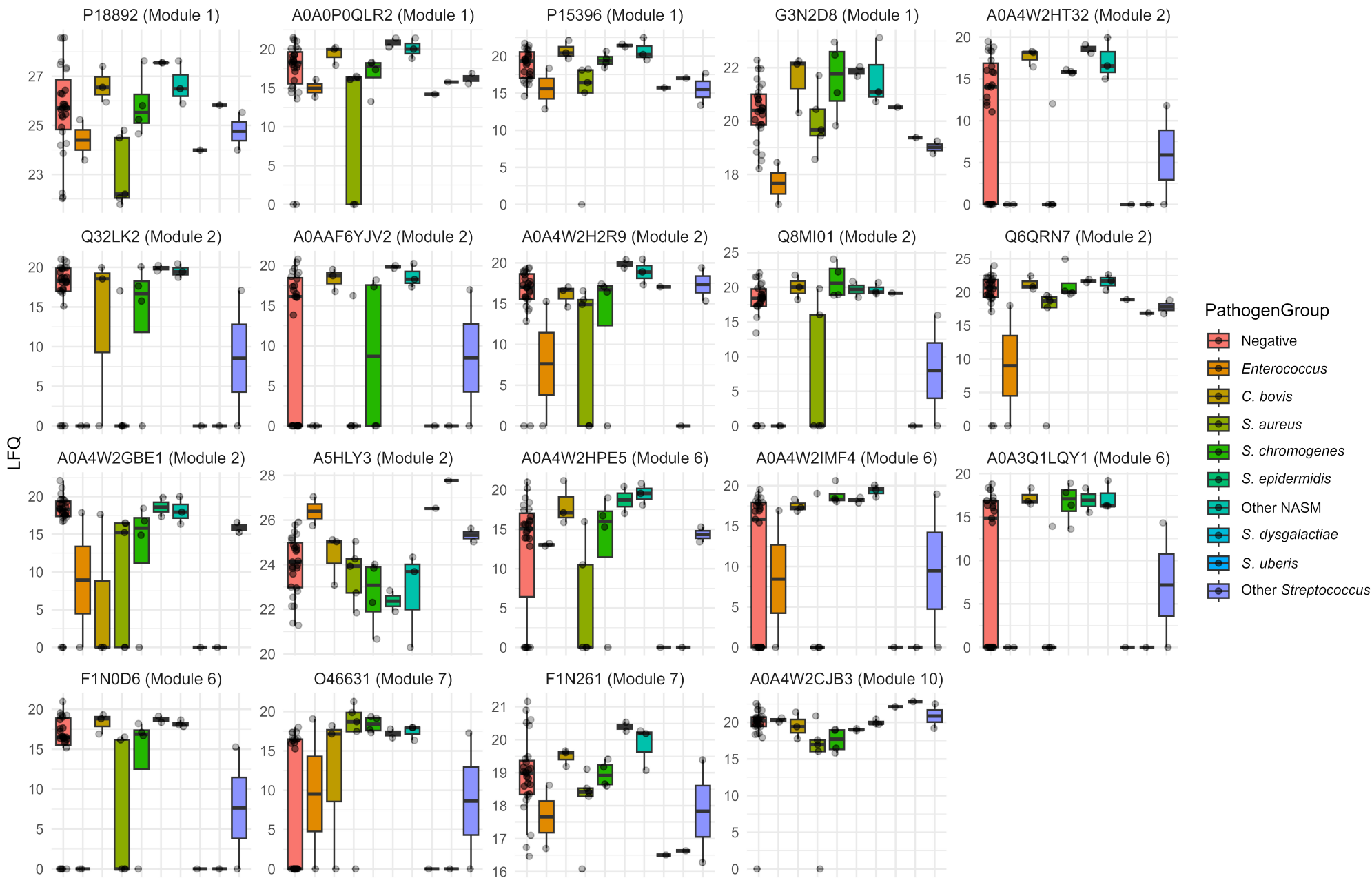

Supplement: Supplementary file 6 — Additional file 6. Proteins involved in immune response with differential expression between pathogen groups. Expression of 19 differently expressed proteins related to immune function. [file 13567_2026_1754_MOESM6_ESM.pdf]
