# Supplementary material for: Bacteria-dependent modulation of immune responses in the bovine udder
Source: Vet Res. 2026 Apr 10;57:75. doi: 10.1186/s13567-026-01754-6 (PMC13181906; doi:10.1186/s13567-026-01754-6)
Supplement: Supplementary file 5 — Additional file 5. Differently expressed proteins between pathogen group. List of 67 differently expressed proteins. [file 13567_2026_1754_MOESM5_ESM.docx]

| **ProteinID** | **Protein name** | **p_value** | **Module** |
| --- | --- | --- | --- |

| A0A4W2GI82 | cGMP-dependent protein kinase | 0.01060 | Module1 |
| --- | --- | --- | --- |
| A0AAF7AKY8 | NADH-cytochrome b5 reductase | 0.01090 | Module1 |
| P15396 | Ectonucleotide pyrophosphatase/phosphodiesterase family member 3 | 0.01380 | Module1 |
| G3N2D8 | Glutathione hydrolase | 0.02300 | Module1 |
| A0A4W2HTA1 | Guanine nucleotide-binding protein G(o) subunit alpha | 0.02570 | Module1 |
| P18892 | Butyrophilin subfamily 1 member A1 (BT) | 0.02710 | Module1 |
| E1BA29 | Guanine nucleotide-binding protein subunit alpha | 0.02870 | Module1 |
| F1N1N6 | Perilipin | 0.02980 | Module1 |
| A9XTK4 | 3-keto-steroid reductase/17-beta-hydroxysteroid dehydrogenase 7 | 0.03090 | Module1 |
| A0A0P0QLR2 | Toll-like receptor 2 | 0.03120 | Module1 |
| Q0VCJ8 | Protein-L-histidine N-pros-methyltransferase | 0.03180 | Module1 |
| Q2KIS4 | Dehydrogenase/reductase (SDR family) member 1 | 0.03330 | Module1 |
| A0A4W2GHH5 | G protein subunit alpha 13 | 0.03480 | Module1 |
| Q9N287 | DnaJ homolog subfamily C member 12 | 0.03490 | Module1 |
| Q05927 | 5'-nucleotidase (5'-NT) | 0.03540 | Module1 |
| A0A4W2D6D0 | Cation-transporting ATPase | 0.03620 | Module1 |
| A0A4W2GND4 | Platelet glycoprotein 4 | 0.03680 | Module1 |
| A0A4W2CTP6 | small monomeric GTPase | 0.03960 | Module1 |
| A0A4W2GYZ4 | Synaptosomal-associated protein | 0.04060 | Module1 |
| Q3SX32 | Perilipin | 0.04110 | Module1 |
| F1MUP9 | Synaptic vesicle membrane protein VAT-1 homolog | 0.04360 | Module1 |
| A0A4W2ISC8 | Xanthine dehydrogenase/oxidase | 0.04940 | Module1 |
| F1MX05 | Saccharopine dehydrogenase-like oxidoreductase | 0.02890 | Module10 |
| Q58CY6 | Prostamide/prostaglandin F synthase | 0.03720 | Module10 |
| A0A4W2CJB3 | Fibrinogen gamma chain | 0.04120 | Module10 |
| Q6R8F2 | Cadherin-1 | 0.04730 | Module10 |
| A0A4W2HL62 | small monomeric GTPase | 0.04770 | Module10 |
| Q2NKV1 | Angiogenin, ribonuclease | 0.04840 | Module10 |
| A0A4W2I9W7 | RAB2A, member RAS onco family | 0.01180 | Module2 |
| Q3T0D7 | Small COPII coat GTPase SAR1A | 0.01550 | Module2 |
| A0A4W2HT32 | Volume-regulated anion channel subunit LRRC8A | 0.01560 | Module2 |
| A0A4W2FZI0 | CD109 antigen | 0.01570 | Module2 |
| Q6QRN7 | PP1201 protein (Responsive to centrifugal force and shear) ( | 0.01910 | Module2 |
| Q32LK2 | Cysteine-rich and transmembrane domain-containing protein 1 | 0.02020 | Module2 |
| A0A4W2CPU3 | Xanthine dehydrogenase/oxidase | 0.02370 | Module2 |
| Q8MI01 | Mucin-15 | 0.02480 | Module2 |
| A0A4W2GBE1 | VPS37B subunit of ESCRT-I | 0.02530 | Module2 |
| A5HLY3 | Lactotransferrin | 0.03050 | Module2 |
| F1MNG3 | small monomeric GTPase | 0.03060 | Module2 |
| A5PK13 | Volume-regulated anion channel subunit LRRC8C | 0.03070 | Module2 |
| Q2TBH2 | RAS related (Related RAS viral (R-ras) oncogene homolog) | 0.03170 | Module2 |
| E1BCI3 | Solute carrier family 7 member 4 | 0.03400 | Module2 |
| A0A4W2DFB2 | InaD-like protein (Protein associated to tight junctions) | 0.03440 | Module2 |
| A0A4W2H2R9 | Polyunsaturated fatty acid lipoxygenase ALOX15 | 0.03590 | Module2 |
| A0A3Q1M9U9 | Solute carrier family 29 (Nucleoside transporters), | 0.03680 | Module2 |
| F1MY82 | Protein kinase C eta type | 0.04010 | Module2 |
| Q2KI18 | Patatin-like phospholipase domain-containing protein 2 | 0.04120 | Module2 |
| A0A4W2IJW1 | Glutathione peroxidase | 0.04290 | Module2 |
| A0A4W2D902 | Protein PALS1 (MAGUK p55 subfamily member 5) (Protein associated with Lin-7 1) | 0.04330 | Module2 |
| A0AAF6YJV2 | Guanine nucleotide-binding protein G(i) subunit alpha-1 | 0.04790 | Module2 |
| Q3MHW6 | Monocarboxylate transporter 1 (MCT 1) | 0.04800 | Module2 |
| A0A4W2C1A3 | Acylglycerol transacylase | 0.04800 | Module2 |
| P02313 | Non-histone chromosomal protein HMG- | 0.04080 | Module4 |
| A0A4W2F6C3 | Surfeit locus protein 4 | 0.01250 | Module6 |
| Q95135 | Excitatory amino acid transporter 3 ( | 0.01570 | Module6 |
| A0A4W2DX83 | ATP synthase peripheral stalk subunit OSCP | 0.01920 | Module6 |
| A0A4W2HPE5 | Multidrug resistance-associated protein 4 | 0.03260 | Module6 |
| A0AAA9S6Z3 | Oxysterol-binding protein | 0.03290 | Module6 |
| B2CZT3 | NAD-dependent protein deacetylase sirtuin-2 | 0.03630 | Module6 |
| A0A4W2IMF4 | PRA1 family protein | 0.03640 | Module6 |
| F1N0D6 | Tyrosine-protein kinase | 0.03790 | Module6 |
| A0A3Q1LQY1 | Ciliary neurotrophic factor receptor subunit alpha | 0.04480 | Module6 |
| L7R5X3 | Elongation factor 1-delta | 0.04910 | Module6 |
| P02316 | Non-histone chromosomal protein HMG-14 | 0.00717 | Module7 |
| O46631 | Tyrosine-protein phosphatase non-receptor type substrate 1 | 0.01690 | Module7 |
| A5PJY0 | LMAN1 protein | 0.03460 | Module7 |
| F1N261 | Tyrosine-protein kinase | 0.04830 | Module7 |
